# Role of Nutrition and Adherence to the Mediterranean Diet in the Multidisciplinary Approach of Hidradenitis Suppurativa: Evaluation of Nutritional Status and Its Association with Severity of Disease

**DOI:** 10.3390/nu11010057

**Published:** 2018-12-28

**Authors:** Luigi Barrea, Gabriella Fabbrocini, Giuseppe Annunziata, Giovanna Muscogiuri, Marianna Donnarumma, Claudio Marasca, Annamaria Colao, Silvia Savastano

**Affiliations:** 1 Dipartimento di Medicina Clinica e Chirurgia, Unit of Endocrinology, Federico II University Medical School of Naples, Via Sergio Pansini 5, 80131 Naples, Italy; giovanna.muscogiuri@gmail.com (G.M.); colao@unina.it (A.C.); sisavast@unina.it (S.S.); 2 Dipartimento di Medicina Clinica e Chirurgia, Unit of Dermatology, Federico II University Medical School of Naples, Via Sergio Pansini 5, 80131 Naples, Italy; gafabbro@unina.it (G.F.); mavabe@hotmail.it (M.D.); claudio.marasca@gmail.com (C.M.); 3 Department of Pharmacy, University of Naples “Federico II”, Italy; Via Domenico Montesano, 49, 80131 Naples, Italy; giuseppe.annunziata@unina.it

**Keywords:** nutrition, body composition, Mediterranean diet, Hidradenitis Suppurativa, phase angle, 7-day food records

## Abstract

Hidradenitis suppurativa (HS) is a chronic, inflammatory and debilitating skin disorder. The exacerbating factors of HS include nutrition and adiposity. We aimed to investigate the relationships between body composition and the adherence to the Mediterranean diet (MD) with the severity of HS in a sample of naive-treatment patients with HS. In this case–controlled, cross-sectional study, we enrolled 41 HS patients and 41 control subjects. Body composition was evaluated by a bioelectrical impedance analysis (BIA) phase-sensitive system. PREvención con DIeta MEDiterránea (PREDIMED) and the 7-day food records were used to evaluate the degree of adherence to the MD and dietary pattern, respectively. The clinical severity was assessed by using the Sartorius HS score. HS patients had a worse body composition, in particular lower phase angle (PhA) (*p* < 0.001), and a lower adherence to the MD than controls, in spite of no differences in energy intake between the two groups. The receiver operator characteristic (ROC) analysis showing a value of PhA of ≤ 5.7 and a PREDIMED score of ≤ 5.0 identified HS patients with the highest clinical severity of the disease. After adjusting for sex, age, body mass index (BMI), and total energy intake, the HS Sartorius score maintained negative correlations with PhA (*p* < 0.001), PREDIMED score, and n-3 polyunsaturated fatty acids (*p* = 0.005). The results of the multivariate analysis showed PhA and PREDIMED score were the major determinants of HS Sartorius score, explaining 82.0% and 30.4% of its variability, respectively (*p* < 0.001). Novel associations were demonstrated between PhA and the degree of adherence to the MD with the HS severity. PhA and PREDIMED score might represent possible markers of severity of HS in a clinical setting.

## 1. Introduction

Hidradenitis suppurativa (HS) or acne inversa, first described by Verneuil’s in 1854 [1], is a chronic inflammatory, debilitating, immune-mediated, suppurative and disabling skin disease, characterized by subcutaneous nodules; its pathophysiology to date is not well understood [2].

Although the real prevalence of HS is still undefined, a prevalence of 1% was reported in the general population in Europe [3,4], which increased up to 4% among young adult women [5]. American Epidemiological studies, reported a prevalence between 0.05% and 0.20% [6,7]. Clinical lesions and chronicity are important to establish the diagnosis of HS, while diagnostic tests to facilitate diagnosis are still lacking [8]. HS affects begin in an individual’s early 20s: It begins after puberty [9] with greater prevalence predominantly in women (3 to 5:1) [10]. In general, the HS leads to follicular occlusion, followed by its rupture resulting in an immune response [11]. Beyond genetic predisposition, several environmental factors including nutrition and adiposity, contribute to the clinical severity of HS phenotype [11]. Obesity is an important risk factor for HS [12]. Several studies report that the severity of HS is positively associated with body mass index (BMI), with rates of obesity that vary from 12% to 88% in HS patients [12]. Nowadays, being overweight or obese is considered to have a leading role in the pathogenesis of HS. In fact obesity induces a subacute inflammatory state, that can increase circulating levels of pro-inflammatory cytokines [8,13]. In particular, the macrophages infiltrating the visceral fat are committed to secrete pro-inflammatory cytokines exacerbating the disease activity of HS [14]. This inflammatory “milieu” of obesity may amplify the effect of the pro-inflammatory cytokines in HS lesions, contributing to overall systemic inflammation [15,16]. In addition, the excess of body weight may exacerbate HS via skin clothing friction [14]. BMI, commonly used as surrogate measurement of adiposity, is an inexpensive and easy method that does not measure either directly the body fat [17], or the body composition [18]. In fact, this limitation of BMI has been recognized by the National Heart, Lung, and Blood Institute (NHLBI) Clinical Guidelines [18]. Therefore, currently waist circumference (WC) is recommended as a surrogate measure of visceral fat distribution [19], which in subjects with obesity represents the main producer of pro-inflammatory cytokines [20]. The association between WC and HS has been reported by several studies [21,22], underlying the role of the pro-inflammatory cytokines produced by visceral fat in the progression and the pathogenesis of HS [23]. Beyond body weight, subjects with HS showed higher body fat than in healthy controls, independent of their BMI [24,25]. Bioelectrical impedance analysis (BIA) is a non-invasive, and reliable tool to estimate body composition that is particularly suitable for studies on large population samples. [26]. Nevertheless, there are only few studies investigating the body composition of HS patients [24,25].

The exacerbating factors of HS include the role of diet [27]. Previous studies have targeted the possible role of dairy products or foods containing brewer’s yeast [28,29]. Nevertheless, in free-living populations, the diet is a highly correlated combination of foods and nutrients, from which it is difficult to extrapolate the effect of a single nutrient or food group from others [30]. The use of 7-day food records is considered as the “gold standard” of self-administered food frequency questionnaires [31]. To date, no studies carefully evaluated, in patients with HS, the dietary intake by 7-day food records, the association of single food components, or the adherence to healthy eating patterns, such as the MD [27]. Nonetheless, the assessment of the nutritional status in the management of HS is a topic of great interest for nutritionist and dermatologist. In the wake of this evidence, we aimed to investigate the relationships between nutritional status, the adherence to the MD, body composition, and the severity of HS in a sample of naive-treatment patients with HS compared to control group matched for sex, age, and BMI.

## 2. Materials and Methods

### 2.1. Design and Setting

This cross-sectional observational study was conducted in patients with HS attending the Unit of Endocrinology, Department of Clinical Medicine and Surgery, University Federico II of Naples (Italy), from January 2017 to April 2018. The study was approved by the Local Ethical Committee (n. 201/15) and carried out in accordance with the Code of Ethics of the World Medical Association (Declaration of Helsinki) for experiments involving humans. The purpose of the protocol was clearly explained to all the study participants, and a written informed consent was obtained. The study was conducted without support from the pharmaceutical industry. This study was registered at clinicaltrials.gov as NCT03683238.

### 2.2. Population Study

The subjects were enrolled in the outpatient Dermatologic and Endocrinology clinics in our Department. The study included 41 treatment-naive patients of both genders out of 63 unselected Caucasian subjects affected by HS attending the Outpatient Clinic of the Unit of Dermatology of the same Department. Forty-one Caucasian healthy subjects (ascertained by participant questionnaire excluding the presence of clinical conditions that potentially influences fluid balance, i.e., myocardial, renal, or endocrine diseases) with BMI ranging 19.2–48.1 kg/m^2^ were chosen as controls among hospital volunteers and employees from the same geographical area around Naples (Italy). Controls were matched by age, sex and BMI and a full medical history, including drug use, was collected. In both groups, all female subjects were assessed during the follicular phase of their menstrual cycle. They did not report being pregnant or to be lactating. To increase the homogeneity of the patient sample we included treatment-naive adult patients only. In particular, the patients were eligible for the study if they had HS diagnosed ≥6 months before study initiation without therapy for at least 3 months. All three diagnostic criteria for HS had to be met: Presence of typical lesions, anatomical sites involved in typical areas and an evolving disease course with relapses and chronicity. While, the exclusion criteria were the following:

1. Occasional or current of use of drugs for HS, including topical antibiotics and systemic treatments (such as clindamycin–rifampicin, tetracycline, Rifampicin–moxifloxacin–metronidazole, ertapenem, acitretin, cyclosporine A, dapsone, isotretinoin, biologics (10 subjects);

2. Specific nutritional pattern or hypocaloric diet in the last three months (two subjects);

3. Supplementation with antioxidants, vitamins or minerals (three subjects);

4. Use of drugs affecting fluid balance, including hormone replacement therapy (four subjects) and non-steroidal anti-inflammatory drugs (three subjects).

**Figure 1:**
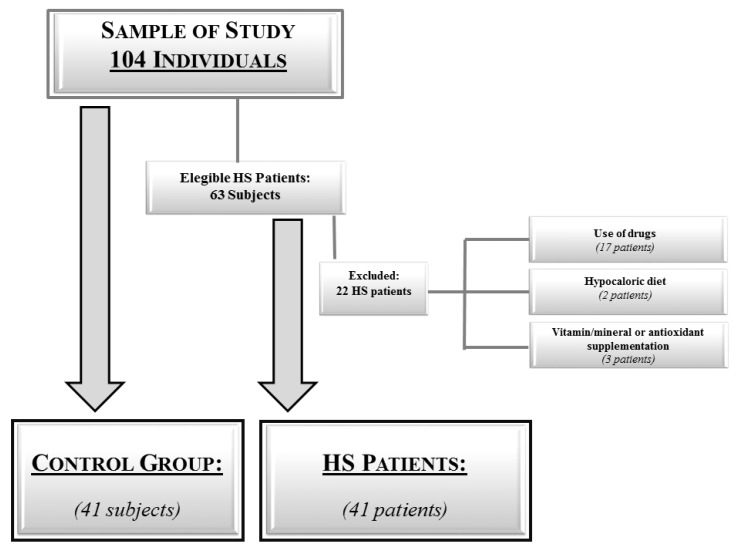
Flow chart of the studied subjects. Abbreviation: HS, Hidradenitis suppurativa.

The flow chart of study subjects is reported in Figure 1. The clinical and biochemical assessment were done between 8 a.m. and 12 p.m., after an overnight fast.

#### Power Size Justification

The power size was calculated based on the following assumptions:

1. In the absence of clinical studies with similar experimental design currently available in the literature, the calculation of the sample size was performed a priori by considering the effect size 0.8 with type I error of 0.05 and a power of 90%. The number of subjects to be enrolled was found to be 34 per group, that we decided to round up to 41, with a total of 82 total subjects enrolled in the study in order to replace drop out patients. The calculation of the Sample Size was performed using G*Power Software (G*power software Version 3.1.9.2, Universität Düsseldorf, Düsseldorf, Germany).

2. The power sample was calculated by the differences of means ± standard deviation (SD) of PhA in each group (6.06). Considering a number of cases required in each group were set at 41 per group, a type I (alpha) error of 0.05 (95%), and a type II (beta) of 0.05 were used, the calculated power size was 95%. The calculation of power size was performed using Sample Size Calculator Clinical Calc (ClinCalc LLC, Chicago, IL, USA).

### 2.3. Lifestyle Habits

We defined current smokers were subjects smoking at least one cigarette per day, former smokers were subjects who had stopped smoking at least one year before the interview, and non-current smokers as the remaining participants. Participants habitually engaged in at least 30 min/day of aerobic exercise (YES/NO) were defined as physically active.

### 2.4. Anthropometric Measurements and Blood Pressure

The anthropometric measurements were performed by the same operator (a nutritionist experienced in providing nutritional assessment and body composition), according to the International Society for the Advancement of Kinanthropometry (ISAK 2006). BMI was calculated as the ratio between weight and square of height (kg/m^2^). WC was measured according to Nishida et al. [32]. Further details are reported in Supplementary data.

### 2.5. Bioelectrical Impedance Analysis

Body composition was assessed using a BIA phase-sensitive system by experienced observers (an 800-µA current at a signal-frequency of 50 kHz BIA 101 RJL, Akern Bioresearch, Florence, Italy) [33], as previously reported [34,35,36,37]. The exam was performed as suggested by the European Society of Parental and Enteral Nutrition (ESPEN) [38]. Electrodes were placed on the hand and the ipsilateral foot, according to Kushner (1992) [39]. The PhA was obtained from conditions under 50 kHz according to the following formula: PhA (°, degrees) = arctangent reactance (Xc)/ resistance (R) × (180/π). Further details are reported in the Supplementary data.

### 2.6. Adherence to the MD

The adherence to the MD was assessed using a PREDIMED questionnaire, consisting of 14 items [40]. This questionnaire, which had already been used in previous studies [36,41,42,43,44,45,46,47,48], was administered by a qualified nutritionist during a face-to-face interview. Briefly, by assigning a score 1 and 0 for each item, PREDIMED score was calculated as follows: 0–5, lowest adherence; score 6–9, average adherence; score ≥10, highest adherence [40].

### 2.7. Dietary Assessment

As we have already fully reported in previous studies [42,43,45,47,48,49], nutritional data was obtained by a face-to-face interview administered by a qualified nutritionist. In detail, a photographic food atlas (≈1000 photographs) of known portion sizes was used to quantify foods and drinks [50] and the 7-day food records were used to collect dietary data, including beverage intakes. On the basis of these records, the nutritionist calculated the total energy intake and the quantities of macronutrients.

### 2.8. Classification and Severity Assessment of HS

As a gold standard is still lacking, the disease severity assessment of HS was assessed using three scoring systems, including Sartorius HS score, Hurley Stages, and HS Physician’s Global Assessment (PGA) [8]. The Sartorius HS score is a clinical classification system based on the counting of single fistulas and nodules within seven anatomical regions [51], and the measurement of the longest distance between two lesions of the same type within each anatomical region (axilla, gluteal, groin, genital, or other inflammatory sites left and/or right) [8]. The Hurley system describes three distinct clinical stages, in particular: “Stage I: Abscess formation, single or multiple, without sinus tracts and cicatrization; stage II: Recurrent abscesses with tract formation and cicatrization, single or multiple, widely separated lesions; and stage III: Diffuse or near-diffuse involvement, or multiple interconnected tracts and abscesses across the entire area”. Finally, according to the HS-PGA HS severity was classified by counting “the number of abscesses, fistulas, and inflammatory and non-inflammatory nodules in all skin areas stages, on a scale from 1 to 6 (from stage 1: Clear, no inflammatory or non-inflammatory nodules to stage 6: Severe, >5 abscesses or draining fistulas)” [52,53]. The dermatologists who evaluated the clinical severity of HS were blinded to the design of the study to prevent rate biases.

### 2.9. Evaluation of the Oxidized Low-Density Lipoprotein Levels

Blood samples were collected via brachial puncture into 5-mL heparin vacuum tubes. The samples were centrifuged at 3000 rpm for 10 min at RT, and sera were separated and stored at −80 °C for further analysis (maximum, 6 months). The plasma levels of oxidized low-density lipoprotein (ox-LDL) were measured by using the LP-CHOLOX test carried out on an automated analyzer (Free Carpe Diem, Diacron International, Grosseto, Italy), using a commercial kit (Diacron International, Grosseto, Italy) according to the manufacturer’s instructions. The LP-CHOLOX test evaluates a class of hydroperoxides derived from the lipid peroxidation, which are mainly represented by oxidized cholesterol. Peroxides are able to promote the oxidation of the ferrous iron (Fe^2+^) to ferric iron (Fe^3+^). The LP-CHOLOX test is based on the spectrophotometric measurement (at 505 nm) of the colored complex developed by the binding between the Fe^3+^ and the thiocyanate. The absorbance values are directly proportional to the lipoperoxides concentrations, and the values are related to specific standard solution (400 μEq/L). Results are expressed in μEq/L, and reference values are: Normal, ≤599 μEq/L; slight alteration, from 600 to 799 μEq/L; moderate alteration, from 800 to 999 μEq/L; strong alteration, ≥1000 μEq/L [54,55].

### 2.10. Statistical Analysis

The data distribution was evaluated by a Kolmogorov-Smirnov test and the abnormal data were normalized by logarithm. Skewed variables were back-transformed for presentation in tables and figures. Results are expressed as mean ± SD. The chi square (χ^2^) test was used to determine the significance of differences in frequency distribution of gender, smoking habit, physical activity, BMI categories, WC cut-offs, and dietary components included in the PREDIMED questionnaire. Differences between HS patients and the control group were analyzed by Student’s paired *t*-test, while the differences among the several parameters with the clinical severity of HS according to the Hurley system and HS-PGA grades were analyzed by Student’s unpaired *t*-test. The correlations between study variables were performed using Pearson *r* correlation coefficients. The association among quantitative variables (Hurley system and HS-PGA grades) with body composition, total energy and daily macronutrients/micronutrients intake were assessed with proportional Odds Ratio (OR) models, 95% Interval Confidence (IC), and *R*^2^. Receiver operator characteristic (ROC) curve analysis which were performed to establish sensitivity and specificity, area under the curve (AUC), and IC, as well as cut-off values for PhA and PREDIMED score in detecting Sartorius HS scores above the median values in the HS patients. Test AUC for ROC analysis was also calculated and we entered 0.957 for AUC ROC and 0.5 for the null hypothesis values. An α level of 0.05 (type 1 error) and a β level of 0.20 (type II error) were used as the cut-off values for statistical significance. Only variables that had a *p*-value < 0.05 in the univariate analysis (partial correlation) were entered. Variables with a variance inflation factor (VIF) >10 were not considered to avoid multicollinearity. Values ≤ 5% were considered statistically significant. Data were collected and analyzed using the MedCalc^®^ package (Version 12.3.0 1993–2012 MedCalc Software bvba—MedCalc Software, Mariakerke, Belgium).

## 3. Results

The study population consisted of 82 participants, 41 patients with HS and 41 healthy subjects as a control group. All case-patients completed the study protocol including nutritional assessment, PREDIMED questionnaire, and BIA measurements. The HS Sartorius score was 51.0 (33.0–81.0). According to the Harley grade and HS-PGA, 14, 24 and 3 patients presented with grade 1, 2 and 3, respectively. Considering the number of grade 3 patients, HS patients with grade 2–3 were included in the same group for the following analysis. These results are shown in the Tables S1–S5.

As reported in Table 1, no significant differences were evident in lifestyle habits and anthropometric measurements between HS patients and healthy subjects.

The body composition evaluated by the BIA parameters of the HS patients and the control group are shown in Table 2. In particular, HS patients had the lowest values of reactance (Xc) (*p* = 0.003), PhA (*p* < 0.001), intra-cellular water (ICW) (%), and the highest values of extra-cellular water (ECW) (*p* < 0.001).

Analyzing the details of the response frequency of dietary components included in the PREDIMED questionnaire, we found that HS patients consumed less frequently red meat and more frequently fish/seafood (Table 3).

Data on Mediterranean food frequencies were further analyzed by using the 7-day food records. As shown in Table 4, in spite of no differences in energy intake between the two groups, HS patients consumed a lower quantity of complex carbohydrate, monounsaturated fatty acids (MUFA) and n-3 polyunsaturated fatty acids (PUFA), and a higher quantity of saturated fatty acid (SFA) and n-6 PUFA than healthy individuals.

**Figure 2:**
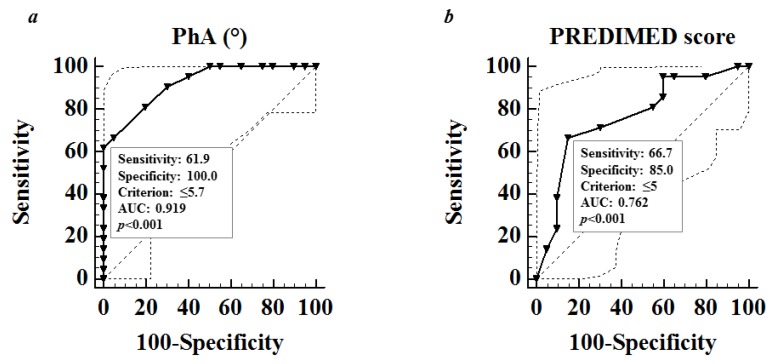
ROC analysis was performed to determine the cut off values of the PhA (**a**) and the PREDIMED score (**b**) that were predictive of the highest Sartorius HS scores (above the median value 51) (Figure 2a,b). A *p* value in bold type denotes a significant difference (*p* < 0.05). ROC, receiver operating characteristic; PhA, phase angle.

ROC analysis was performed to determine the cut off values of the PhA and the adherence to the MD that were predictive of highest Sartorius HS scores (above the median value 51) (Figure 2a,b). A value of PhA of ≤5.7 (*p* < 0.001, AUC 0.919, standard error 0.040, 95% CI 0.790 to 0.981) and a score of PREDIMED score of ≤5.0 (*p* < 0.001, AUC 0.762, standard error 0.077, 95% CI 0.603 to 0.881) could serve as a thresholds for a significantly increased risk of high Sartorius HS scores.

### Correlation Studies

The correlations between HS Sartorius score and body composition evaluated by BIA parameters are summarized in Table 5. Also, after adjusting for sex, age, BMI, and total energy intake, HS Sartorius score showed significant negative correlations with PhA (*p* < 0.001), but not with R and Xc, fat mass (FM) (*p* = 0.001), ICW (*p* < 0.001), and had positive associations with free fat mass (FFM) (%), and ECW (*p* = 0.004).

**Figure 3:**
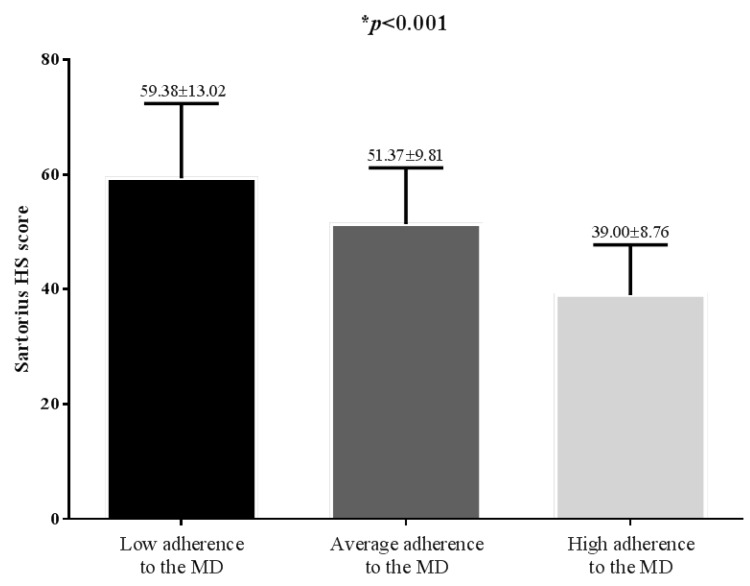
Differences in HS Sartorius scores across the PREDIMED categories. Higher HS Sartorius scores were evidenced in low adherers compared with average-higher adherers (*p* < 0.001).

In Figure 3 we show the differences in HS Sartorius scores across the PREDIMED categories. Higher values of HS Sartorius scores were evidenced in low adherers, compared with average-higher adherers (*p* < 0.001).

**Figure 4:**
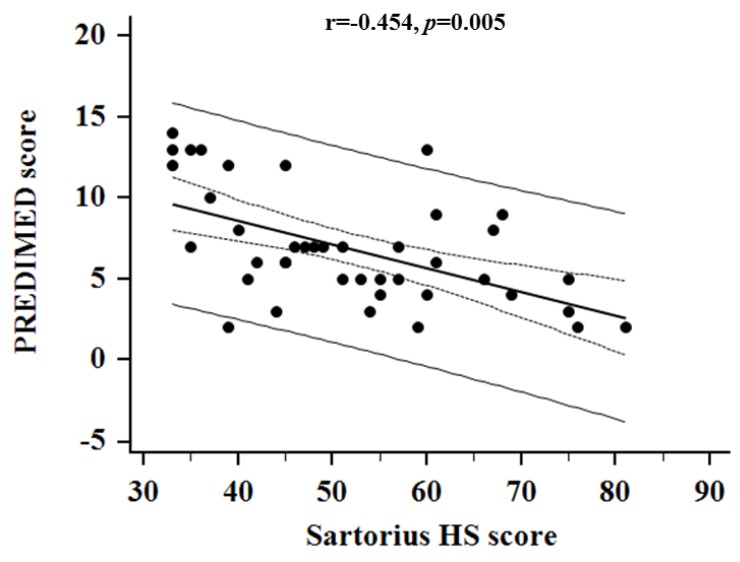
Correlation between HS Sartorius score and PREDIMED score, after adjusting for sex, age, BMI and total energy intake. The highest HS Sartorius score was significantly associated with the lowest score of PREDIMED score (*r* = –0.552, *p* < 0.001), and this correlation remained significant also after adjusting for sex, age, BMI and total energy intake (*r* = –0.454, *p* = 0.005). A *p* value in bold type denotes a significant difference (*p* < 0.05).

Consequently, the highest HS Sartorius score was significantly associated with lowest score of PREDIMED score (*r* = –0.552, *p* < 0.001), and this correlation remained significant also after adjusting for sex, age, BMI and total energy intake (*r* = –0.454, *p* = 0.005), as showed in Figure 4.

In Table 6 we show the correlations among HS Sartorius scores, total energy and daily macronutrients intake evaluated by using the 7-day food records. The HS Sartorius score showed highly positive correlations with total and simple carbohydrate and a negative correlation with n-3 PUFA; however, after adjusting for sex, age, and BMI only the negative correlation with n-3 PUFA remained significant. After adjusting for sex, age, BMI and total energy intake, ox-LDL levels were positively correlated with the HS Sartorius score (*r* = 0.436, *p* = 0.007), and negatively with PhA (*r* = −0.506, *p* = 0.001) and PREDIMED score (*r* = −0.701, *p* < 0.001). Similar correlations were also found by using the Harley grade severity of HS and the data are reported in the supplementary files.

To evaluate the relative prognostic value of the measures of body composition and nutritional status to predict the clinical severity of HS, we performed two multiple linear regression analysis models that included measures of the body composition parameters (model I), PREDIMED scores, n-3 PUFA and ox-LDL (model II). Using model I, PhA entered at the first step (*p* < 0.001) and appeared to be among the body composition measures exerting the most powerful influence on HS Sartorius scores, explaining 82.0% of HS Sartorius score variability, while the other variables (FM, FFM, ECW, ICW) were excluded. Using model II, PREDIMED scores were entered at the first step (*p* < 0.001) and seemed to be the most powerful factor influencing the HS Sartorius score, explaining 30.4% of HS Sartorius score variability, while the other variables (while n-3 PUFA and ox-LDL) were excluded. The results of the two models are shown in Table 7.

## 4. Discussion

The novel findings of this cross-sectional, case-controlled, observational study are the associations between the clinical severity of HS with PhA, a marker of cell membrane integrity, and with nutritional status, in particular the degree of adherence to the MD. Our data demonstrates that PhA was smaller and the adherence to the MD was lower in HS patients than in control subjects. Moreover, both the associations were independent of gender, age, BMI, and total energy intake. Based on ROC curve analysis, PhA ≤ 5.7° and a PREDIMED score of ≤ 5.0 identified HS patients who presented with the highest clinical severity of the disease. Consistently, we found that HS patients presented significant differences in other BIA parameters indicative of an inflammatory/catabolic status and exhibited a pro-inflammatory dietary pattern, with a lower intake of complex carbohydrate, MUFA and n-3 PUFA, and a higher intake of SFA and n-6 PUFA than controls.

HS is a chronic, immune-mediated inflammatory skin disease with a multifactorial pathogenesis. HS develops through interplay of genetic, immunological, endocrine and environmental risk factors, including obesity [12], body composition [24,25], and diet [27]. In particular, both Romaní et al. and Miller et al. reported that subjects with HS and healthy controls showed differences in FM analyzed by BIA, irrespective of their BMI, with higher FM in HS patients than in healthy controls [24,25]. In this context, higher FM may indicate an altered status of inflammatory homeostasis in adipose tissue that can amplify the morbidity in HS patients. Nevertheless, as a BIA volumetric approach provides an indirect estimate of FM, FM could be both over- and under-estimated due to minimal variations of soft tissues hydration that might induce errors in the equations used in conventional BIA, for the prediction of body composition [56,57]. Contrariwise, the PhA, which is derived from raw BIA measurements such as R and Xc, is considered to be valid in conditions with altered hydration status, as in chronic skin inflammation, such as psoriasis, obesity, and chronic inflammation [35,58,59] and the advantages of using the raw BIA measurements, including PhA, have been also recently emphasized [60]. In both these studies, PhA was not included among the BIA parameters of body composition, and the nutritional assessment was not performed.

PhA, a parameter obtained from BIA direct measures, such as R and Xc, is widely used as a marker of cellular health [61]. Consequently, PhA is considered a reliable predictor of mortality and morbidity in several diseases [58]. In a healthy population, a number of different factors may affect PhA, including diet, sex, age, BMI, and inflammatory status [62]. PhA indicates the integrity [63] of a large number of cell membranes and the water distribution in body fluids [64]. Thus, PhA is positively associated with the body cell mass [65] and negatively associated with ECW/ICW ratio [66]. In line with other studies in different chronic inflammatory diseases [58], and our previous study in psoriatic patients [35], in this study we evidenced that a small PhA were correlated also with the clinical severity of HS, and we hypothesized the inflammatory milieu could account for this association in our group of HS patients. Of interest, among all BIA measurements, PhA was the main predictor of the clinical severity of HS, while R and Xc showed no correlations after adjusting for sex, age, BMI and total energy intake, likely due to the link of PhA with either the capacitive behavior of tissues (Xc) associated with cellularity, cell size, and integrity of the cell membrane, and its pure resistive behavior (R), mainly dependent on tissue hydration [19]. The identification of prognostic factors for HS patients could play a critical role in both the clinical management and the adequate monitoring of the clinical course of the disease.

As in psoriatic patients, we found that HS patients, compared to the control group, had also a lower adherence to the MD. In addition, by carefully evaluating their dietary assessment, HS patients consumed higher amounts of simple carbohydrates, total fat, food with a higher n-6/n-3 PUFAs ratio, and lower complex carbohydrates, MUFA, n-3 PUFA and fiber. The association of diet with the clinical severity of HS has been previously reported. In particular, Danby investigated to the possible role of dairy food on HS disease and it reported that 83% of the 47 patients that underwent a dairy-free diet clinically improved compared to the control group without diet restriction [28]. In addition, Cannistrà et al. reported that in 12 patients with HS, a controlled brewer’s yeast-free diet for 12 months was effective in reducing the clinical severity of HS with a rapid stabilization of the dermatologic manifestation [29].

Very recently, we reported in healthy subjects, an association between the degree of the adherence to the PhA and MD, independently of sex, age, and BMI [34]. This association has been attributed to the high content of different beneficial compounds, such as antioxidants and polyphenols, found in plant foods, fruit and red wine, which are largely present in Mediterranean foods [67]. In particular, Mediterranean meal plans exhibit anti-inflammatory potential based on ox-LDL levels [68], a marker of oxidative stress, closely involved in the process of chronic inflammation. Consistently, in our sample of HS patients, ox-LDL levels presented a negative correlation the adherence to the MD and a positive correlation with the clinical severity of HS, thereby supporting the potential role of the MD in the integrated management of the HS patients.

The main limitations of the study were the following: First, the cross-sectional design of this study did not allow the determination of a causal association between MD and HS or to draw a final conclusion on the prognostic value of the degree of adherence to the MD in the prediction of clinical severity of HS. Further, there was not a normal weight control group matched by age and gender which did not allow a more consistent analysis of the associations detected in this study. Although body weight could have influenced the nutritional behavior in this study, the clinical severity of HS with PhA was associated with the degree of adherence to the MD after correction for BMI. Moreover, the cut-off value of the PhA and of PREDIMED scores suggested in our present study for predicting the clinical severity of HS, should be considered with caution until large studies are available to perform an appropriate cross-validation. In addition, although in this study we based the analysis on raw BIA measurements rather than BIA volumetric parameters, the evaluation of FFM and FM was not validated by gold standard reference methods, such as dual-X ray absorptiometry, and expert nutritionists are mandatory for execution and especially for the assessment of the nutritional status and the interpretation of BIA measurements, in particular PhA. Nevertheless, considering the low prevalence of HS [3,4,5,6,7], strengths of our study include the relatively large sample of HS patients and the diagnosis of HS that was clinically evaluated and not self-reported; moreover, this study has adequate statistical power, included only naive-treatment patients and both HS patients and matched controls have been well characterized. Finally, the nutritional status has been adequately assessed by using the 7-day food records, the gold standard among food frequency questionnaires [31].

## 5. Conclusions

In conclusion, we reported a novel association between the clinical severity of HS with PhA, a marker of cell membrane integrity, and with nutritional status, in particular the degree of adherence to the MD. Specific cut-off values for the PhA and the degree of adherence to the MD could be included as an auxiliary tool in the complex dermatological evaluation of the clinical severity in patients with HS, contributing to identify those patients who could get additional benefit from careful dietary interventions. Our study underlines the role of the nutrition assessment as a predictive tool in HS patients. A growing cooperation between nutritionists and dermatologist might provide a combination key in the complex management of the HS patients. Further studies on a large population with HS and intervention trials are warranted to support the association of PhA and the adherence to the MD with the clinical severity of HS, and to highlight the potential anti-inflammatory effects of the MD on in HS patients.

## Figures and Tables

**Table 1:** Differences in lifestyle habits and anthropometric characteristic in HS patients and healthy subjects.

Parameters	HS Patients *n* = 41	Control Group *n* = 41	*p*-Value
**Lifestyle Habits**			
Gender, male (*n*, %)	14, 34.1%	14, 34.1%	χ^2^ = 0.071, *p* = 0.789
Age (years)	26.22 ± 9.88	27.07 ± 8.06	0.298
Smoking (yes)	22, 53.7%	21, 51.2%	χ^2^ = 0.00, *p* = 1.00
Physical activity (yes)	16, 39.0%	15, 36.6%	χ^2^ = 0.00, *p* = 1.00
**Anthropometric Measurement**			
Weight (kg)	84.97 ± 21.33	84.24 ± 19.46	0.543
Height (m)	1.65 ± 0.09	1.65 ± 0.07	1.000
BMI (kg/m^2^)	31.05 ± 7.66	30.88 ± 7.01	0.750
Normal-weight (*n*, %)	7, 17.1%	10, 24.4%	χ^2^ = 0.30, *p* = 0.586
Overweight (*n*, %)	14, 34.1%	11, 26.8%	χ^2^ = 0.23, *p* = 0.631
Grade I obesity (*n*, %)	12, 29.3%	10, 24.4%	χ^2^ = 0.06, *p* = 0.803
Grade II obesity (*n*, %)	4, 9.8%	6, 14.6%	χ^2^ = 0.011, *p* = 0.736
Grade III obesity (*n*, %)	4, 9.8%	4, 9.8%	χ^2^ = 0.14, *p* = 0.710
WC male (cm)	101.62 ± 15.86	94.52 ± 19.19	0.215
<cut-off (*n*, %)	7, 50%	8, 57.1%	χ^2^ = 0.00, *p* = 1.00
>cuf-off (*n*, %)	7, 50%	6, 42.9%	
WC female (cm)	92.73 ± 19.05	95.11 ± 16.97	0.590
<cut-off (*n*, %)	11, 40.7%	10, 37.0%	χ^2^ = 0.00, *p* = 1.00
>cuf-off (*n*, %)	16, 59.3%	17, 63.0%	

No significant differences in lifestyle habits and anthropometric measurements were evident between the two groups. A *p* value in bold type denotes a significant difference (*p* < 0.05). HS, Hidradenitis Suppurativa.

**Table 2:** Body composition evaluated by BIA parameters of the HS patients and the control group.

BIA Parameters	HS Patients *n* = 41	Control Group *n* = 41	
	Mean ± SD	Mean ± SD	*p* Value
R (Ω)	486.85 ± 73.06	500.29 ± 65.96	0.442
Xc (Ω)	52.15 ± 9.60	58.54 ± 7.82	**0.003**
PhA (°)	6.06 ± 0.76	6.7 ± 0.67	**<0.001**
FM (kg)	30.61 ± 16.90	29.79 ± 16.34	0.938
FM (%)	33.35 ± 11.63	33.05 ± 12.61	0.842
FFM (kg)	55.72 ± 8.52	54.45 ± 6.87	0.350
FFM (%)	65.67 ± 11.98	66.95 ± 12.61	0.504
TBW (Lt)	40.68 ± 6.23	39.86 ± 5.03	0.410
TBW (%)	48.66 ± 8.52	49.01 ± 9.23	0.756
ECW (Lt)	18.65 ± 3.36	17.07 ± 2.39	**<0.001**
ECW (%)	45.79 ± 3.57	42.85 ± 2.80	**<0.001**
ICW (Lt)	22.02 ± 3.56	22.79 ± 3.17	0.224
ICW (%)	54.20 ± 3.57	57.16 ± 2.81	**<0.001**

HS patients exhibited statistically significant differences compared with the control group sex, age and BMI-matched for BIA parameters. In particular HS patients had the lowest values of Xc, PhA, ICW and the highest values of ECW. A *p* value in bold type denotes a significant difference (*p* < 0.05). FM, fat mass; FFM, free fat mass; TBW, total body water; ECW, extracellular water; ICW, intracellular water; BIA, bioelectrical impedance analysis.

**Table 3:** Response frequency of dietary components included in the PREDIMED questionnaire in the HS patients and the control group.

Questions PREDIMED Questionnaire	HS Patients *n* = 41		Control Group *n* = 41			
	*n*	%	*n*	%	χ^2^	*p*-Value
Use of extra virgin olive oil as main culinary lipid	38	92.7	40	97.6	0.26	0.608
Extra virgin olive oil > 4 tablespoons	25	61.0	31	75.6	1.41	0.235
Vegetables ≥ 2 servings/day	14	34.1	17	41.5	0.207	0.649
Fruits ≥ 3 servings/day	18	43.9	26	63.4	2.41	0.121
Red/processed meats < 1/day	18	43.9	31	75.6	7.30	**0.007**
Butter, cream, margarine < 1/day	22	53.7	25	61.0	0.20	0.655
Soda drinks < 1/day	17	41.5	20	48.8	0.19	0.657
Wine glasses ≥ 7/week	11	26.8	14	34.1	0.23	0.631
Legumes ≥ 3/week	23	56.1	19	46.3	0.44	0.508
Fish/seafood ≥ 3/week	16	39.0	31	75.6	9.77	**0.001**
Commercial sweets and confectionery ≤2 /week	18	43.9	14	34.1	0.46	0.497
Tree nuts ≥ 3/week	19	46.3	24	58.5	0.78	0.376
Poultry more than red meats	24	58.5	21	51.2	0.20	0.657
Use of sofrito sauce ≥2 /week	20	48.8	19	46.3	0.00	1.00

In HS patients, extra virgin olive oil was the most consumed food item, followed by poultry more than red meats. HS patients exhibited statistically significant differences in red meats and fish consumption, compared with healthy subjects, A *p* value in bold type denotes a significant difference (*p* < 0.05).

**Table 4:** Total energy and daily macronutrients/micronutrients intake of HS patients and the control group.

Parameters	HS Patients *n* = 41	Control Group *n* = 41	*p*-Value
Total energy (kcal)	2281.49 ± 269.81	2302.68 ± 168.63	0.443
Protein (gr of total kcal)	100.21 ± 17.63	101.54 ± 11.77	0.587
Carbohydrate (gr of total kcal)	310.21 ± 39.44	314.19 ± 24.29	0.391
Complex (gr of total kcal)	208.53 ± 28.30	216.02 ± 18.22	**0.043**
Simple (gr of total kcal)	101.68 ± 13.53	98.18 ± 9.53	0.111
Fat (gr of total kcal)	71.09 ± 8.90	71.08 ± 6.39	0.996
SFA (gr of total kcal)	24.73 ± 3.16	21.81 ± 2.30	**<0.001**
MUFA (gr of total kcal)	32.25 ± 4.74	33.79 ± 2.99	**0.030**
PUFA (gr of total kcal)	14.11 ± 2.57	15.47 ± 2.55	**0.016**
n-6 PUFA (gr/day)	5.81 ± 2.72	4.62 ± 1.24	**0.008**
n-3 PUFA (gr/day)	8.30 ± 1.69	10.85 ± 1.68	**<0.001**

HS patients had the lowest intake of complex carbohydrate, MUFA and n-3 PUFA, and a higher intake of SFA and n-6 PUFA compared to healthy subjects. A *p* value in bold type denotes a significant difference (*p* < 0.05).

**Table 5:** Correlation among body composition evaluated by BIA parameters and Sartorius HS score in HS patients adjusted for sex, age, BMI and total energy intake.

BIA Parameters	Simple Correlations *n* = 41		After Adjusted for Sex, Age, BMI and Total Energy Intake	
	*r*	*p* Value	*r*	*p* Value
R (Ω)	−0.308	**0.050**	0.008	0.961
Xc (Ω)	−0.095	0.553	0.114	0.501
PhA (°)	−0.905	**<0.001**	−0.897	**<0.001**
FM (kg)	0.023	0.886	0.531	**0.001**
FM (%)	0.017	0.915	0.293	0.079
FFM (kg)	−0.240	0.130	−0.088	0.606
FFM (%)	−0.102	0.526	−0.334	**0.044**
TBW (Lt)	0.236	0.137	0.105	0.535
TBW (%)	−0.020	0.900	−0.282	0.091
ECW (Lt)	0.581	**<0.001**	0.463	**0.004**
ECW (%)	0.844	**<0.001**	0.837	**<0.001**
ICW (Lt)	−0.136	0.398	−0.508	**0.001**
ICW (%)	−0.844	**<0.001**	−0.837	**<0.001**

Sartorius HS score was significantly associated with R, PhA, ECW and ICW. All parameters remain significantly associated after adjuster for sex, age, BMI and total energy intake, except for R. A *p* value in bold type denotes a significant difference (*p* < 0.05). R, resistance; PhA, phase angle; ECW, extracellular water; ICW, intracellular water.

**Table 6:** Correlation among total energy, daily macronutrients/micronutrients intake and Sartorius HS score in HS patients.

Parameters	Simple Correlations *n* = 41		After Adjusted for Sex, Age, BMI and Total Energy Intake	
	R	*p*-Value	*r*	*p*-Value
Total energy (kcal)	0.273	0.085	−0.060	0.719
Protein (gr of total kcal)	0.148	0.357	−0.160	0.339
Carbohydrate (gr of total kcal)	0.322	**0.040**	0.111	0.507
Complex (gr of total kcal)	0.280	0.077	0.026	0.875
Simple (gr of total kcal)	0.353	**0.024**	0.201	0.227
Fat (gr of total kcal)	0.155	0.334	−0.114	0.496
SFA (gr of total kcal)	0.193	0.226	−0.089	0.598
MUFA (gr of total kcal)	0.151	0.347	−0.103	0.536
PUFA (gr of total kcal)	0.020	0.902	−0.070	0.674
n-6 PUFA (gr/day)	−0.005	0.976	−0.062	0.711
n-3 PUFA (gr/day)	−0.350	**0.025**	−0.342	**0.035**

Sartorius HS score was significantly associated with total/simple carbohydrate and n-3 PUFA. After adjuster for sex, age, BMI and total energy intake, only n-3 PUFA remain negatively associated with Sartorius HS score. A *p* value in bold type denotes a significant difference (*p* < 0.05).

**Table 7:** Multiple regression analysis models (stepwise method) with the Sartorius HS score as the dependent variable to estimate the predictive value of: BIA parameters (Model I), PREDIMED score, n-3 PUFA and ox-LDL (Model II).

Parameters	Multiple Regression Analysis			
Model 1	***r*^2^**	***β***	***t***	***p* value**
**PhA**	0.820	–0.905	–13.33	**<0.001**
Variable excluded: FM, FFM, ECW, ICW				
Model 2	***r*^2^**	***β***	***t***	***p* value**
**PREDIMED Score**	0.304	–0.552	–4.13	**<0.001**
Variable excluded: n-3 PUFA and ox-LDL				

Among some BIA parameters (model I), PREDIMED scores, n-3 PUFA and ox-LDL (model II), Sartorius HS scores were well predicted by PhA and PREDIMED scores, respectively. A *p* value in bold type denotes a significant difference (*p* < 0.05).

## Supplementary Materials

The following are available online at http://www.mdpi.com/2072-6643/11/1/57/s1, Supplementary data; Table S1: Differences in the Hurley system and HS-PGA grades in BIA parameters; Table S2: Bivariate proportional odds ratio model to assess the association between body composition evaluated by BIA parameters, the Hurley system and HS-PGA grades in HS patients; Table S3: Differences in the Hurley system and HS-PGA grades in the adherence to the MD and Ox-LDL in HS patients; Table S4: Differences of total energy and daily macronutrient/micronutrient intake of HS patients, divided according to the Hurley system and HS-PGA grade; Table S5: Bivariate proportional odds ratio model to assess the association between total energy and daily macronutrient/micronutrient intake of HS patients, divided according to the Hurley system and HS-PGA grade.

## Abbreviations

HS	Hidradenitis suppurativa
MD	Mediterranean diet
BIA	Bioelectrical Impedance Analysis
PREDIMED	PREvención con DIeta MEDiterránea
PhA	phase angle
BMI	body mass index
WC	waist circumference
SD	Standard Deviation
R	resistance
Xc	reactance
GA	Physician’s Global Assessment
ox-LDL	oxidized low-density lipoprotein
OR	Proportional Odds Ratio
IC	interval confidence
ROC	receiver operator characteristic
AUC	area under the curve
ICW	Intra-cellular Water
ECW	Extra-cellular Water
FM	Fat Mass
MUFA	Monounsaturated Fatty Acids
PUFA	Polyunsaturated Fatty Acids
SFA	Saturated Fatty Acid
FFM	Free Fat Mass
