# Supplementary material for: Role of Nutrition and Adherence to the Mediterranean Diet in the Multidisciplinary Approach of Hidradenitis Suppurativa: Evaluation of Nutritional Status and Its Association with Severity of Disease
Source: Nutrients. 2018 Dec 28;11(1):57. doi: 10.3390/nu11010057 (PMC6356593; doi:10.3390/nu11010057)
Supplement: Supplementary file 1 [file nutrients-11-00057-s001.zip › nutrients-406904-supplementary/Supplementary data.docx]

**MATERIALS/SUBJECTS AND METHODS:**

*Anthropometric measurements*

The enrolled subjects wore only light clothes and no shoes. Height was measured to the nearest 0.5 cm using a wall-mounted stadiometer (Seca 711; Seca, Hamburg, Germany). Body weight was determined to the nearest 0.1 kg using a calibrated balance beam scale (Seca 711; Seca, Hamburg, Germany). BMI was classified according to WHO’s criteria as normal weight: 18.5–24.9 kg/m^2^; overweight, 25.0–29.9 kg/m^2^; grade I obesity, 30.0–34.9 kg/m^2^; grade II obesity, 35.0–39.9 kg/m^2^; grade III obesity ≥40.0 kg/m^2^.

WC was measured to the nearest 0.1 cm with a non-stretchable measuring tape placing the meter at the midpoint between the top of the iliac crest and the lower margin of the last palpable rib in midaxillary line.

*Bioelectrical Impedance Analysis*

When the participants performed the exam, they were supine with limbs slightly spread apart from the body, refrained from eating, drinking, and exercising for six hours and no alcohol within 24 h before testing according to the European Society of Parenteral and Enteral Nutrition (ESPEN) guidelines. Subjects had no shoes and socks and contact areas were scrubbed with alcohol immediately before electrode placement. Electrodes (BIATRODES Akern Srl; Florence, Italy) were placed proximal to the phalangeal–metacarpal joint on the dorsal surface of the right hand and distal to the transverse arch on the superior surface of the right foot. Sensor electrodes were placed at the midpoint between the distal prominence of the radius and ulna of the right wrist, and between the medial and lateral malleoli of the right ankle [38]. To avoid interobserver and interdevice variability, all measurements were performed under strictly standardized conditions by the same nutritionist using the same device. The instrument was routinely checked with resistors and capacitors of known values. Reliability for within-day and between-day measurements by the same observer were <3% for resistance (R), <2.8% for reactance (Xc), and <3.1% for R, <2.5% for Xc, respectively. The coefficient of variation (CV) of repeated measurements of R and Xc at 50 kHz was assessed in 10 patients (5 males and 5 females) by the same observer: CVs were 2.8% and 1.9% for R and Xc, respectively.
