# Supplementary material for: Role of Nutrition and Adherence to the Mediterranean Diet in the Multidisciplinary Approach of Hidradenitis Suppurativa: Evaluation of Nutritional Status and Its Association with Severity of Disease
Source: Nutrients. 2018 Dec 28;11(1):57. doi: 10.3390/nu11010057 (PMC6356593; doi:10.3390/nu11010057)
Supplement: Supplementary file 1 [file nutrients-11-00057-s001.zip › nutrients-406904-supplementary/Supplementary Tables.docx]

**Table S1.** Differences in the Hurley system and HS-PGA grades in the BIA parameters.

| BIA parameters | Grade 1  n=14 | Grade 2/Grade 3  n=27 | *p* value |
| --- | --- | --- | --- |
| R (Ω) | 500.15 ± 94.51 | 479.96 ± 60.02 | 0.409 |
| Xc (Ω) | 52.43 ± 11.53 | 52.00 ± 8.68 | 0.894 |
| PhA (°) | 6.72 ± 0.57 | 5.72 ± 0.61 | <0.001 |
| FM (kg) | 30.35 ± 18.32 | 30.75 ± 16.47 | 0.944 |
| FM (%) | 32.19 ± 11.59 | 33.96 ± 11.83 | 0.648 |
| FFM (kg) | 56.83 ± 10.08 | 55.14 ± 7.73 | 0.552 |
| FFM (%) | 64.95 ± 12.67 | 66.04 ± 11.83 | 0.787 |
| TBW (Lt) | 41.54 ± 7.43 | 40.23 ± 5.61 | 0.530 |
| TBW (%) | 49.56 ± 8.39 | 48.20 ± 8.70 | 0.631 |
| ECW (Lt) | 17.90 ± 3.35 | 19.04 ± 3.37 | 0.313 |
| ECW (%) | 43.16 ± 2.80 | 47.16 ± 3.16 | <0.001 |
| ICW (Lt) | 23.64 ± 4.41 | 21.19 ± 2.76 | 0.035 |
| ICW (%) | 56.84 ± 2.80 | 52.83 ± 3.16 | <0.001 |

HS patients with grade 1 *vs* grade 2 in the Hurley system and HS-PGA, had the highest values of PhA, ICW and the lowest values of ECW. Skewed variables were back-transformed for presentation in table. Results are expressed as mean ± standard deviation. Differences between groups were analyzed by unpaired Student’s *t* test. A *p* value in bold type denotes a significant difference (*p <0.05*).

**Abbreviations:** HS, Hidradenitis suppurativa*;* HS-PGA, Hidradenitis suppurativa-Physician’s Global Assessment; R, Resistance (Ω=ohm); Xc, Reactance (Ω=ohm); PhA, Phase Angle (°=degrees); FM, Fat Mass; FFM, Fat-Free Mass; TBW, Total Body Water; ECW, Extra-Cellular Water; ICW, Intra-Cellular Water.

**Table S2.** Bivariate proportional odds ratio model to assess the association between body composition evaluated by BIA parameters , the Hurley system and HS-PGA grade in HS patients.

| Parameters | Grade 1  n=14 | | | | Grade 2/Grade 3  n=27 | | | |
| --- | --- | --- | --- | --- | --- | --- | --- | --- |
|  | **OR** | ***p***  **value** | **95% IC** | **R^2^** | **OR** | ***p***  **value** | **95% IC** | **R^2^** |
| R (Ω) | 1.004 | 0.400 | 0.995 – 1.013 | 0.017 | 0.996 | 0.400 | 0.987 – 1.005 | 0.017 |
| Xc (Ω) | 1.005 | 0.891 | 0.939 – 1.076 | 0.000 | 0.995 | 0.891 | 0.930 – 1.065 | 0.000 |
| PhA (°) | 41.890 | **0.002** | 3.828 – 45.84 | 0.430 | 0.024 | **0.002** | 0.002 – 0.261 | 0.430 |
| FM (kg) | 0.999 | 0.942 | 0.961 – 1.1038 | 0.000 | 1.001 | 0.942 | 0.963 – 1.041 | 0.000 |
| FM (%) | 0.987 | 0.640 | 0.932 – 1.044 | 0.005 | 1.014 | 0.640 | 0.958 – 1.073 | 0.005 |
| FFM (kg) | 1.024 | 0.543 | 0.949 – 1.105 | 0.009 | 0.977 | 0.543 | 0.905 – 1.054 | 0.009 |
| FFM (%) | 0.992 | 0.781 | 0.940 – 1.048 | 0.002 | 1.008 | 0.781 | 0.954 – 1.064 | 0.002 |
| TBW (Lt) | 1.035 | 0.521 | 0.932 – 1.149 | 0.010 | 0.966 | 0.521 | 0.871 – 1.073 | 0.010 |
| TBW (%) | 1.020 | 0.624 | 0.943 – 1.102 | 0.006 | 0.981 | 0.624 | 0.908 – 1.060 | 0.006 |
| ECW (Lt) | 0.895 | 0.309 | 0.724 – 1.108 | 0.027 | 1.117 | 0.309 | 0.903 – 1.382 | 0.027 |
| ECW (%) | 0.552 | **0.003** | 0.371 – 0.821 | 0.328 | 1.812 | **0.003** | 1.219 – 2.695 | 0.328 |
| ICW (Lt) | 1.230 | **0.047** | 1.003 – 1.510 | 0.104 | 0.813 | **0.047** | 0.662 – 0.997 | 0.104 |
| ICW (%) | 1.812 | **0.003** | 1.219 – 2.695 | 0.328 | 0.552 | **0.003** | 0.371 – 0.821 | 0.328 |

The Hurley system and HS-PGA grades were associated with the highest OR of PhA, ECW and ICW. A *p* value in bold type denotes a significant difference (*p <0.05*).

**Abbreviations:** HS, Hidradenitis suppurativa*;* HS-PGA, Hidradenitis suppurativa-Physician’s Global Assessment; R, Resistance (Ω=ohm); Xc, Reactance (Ω=ohm); PhA, Phase Angle (°=degrees); Na/K, Sodium/Potassium; BMR, Basal Metabolic Rate; BCMI, Body Cell Mass Index; FM, Fat Mass; FFM, Fat-Free Mass; BCM, Body Cell Mass; SMM Skeletal Muscle Mass; TBW, Total Body Water; ECW, Extra-Cellular Water; ICW, Intra-Cellular Water; *OR*, Odds Ratio; *IC*, Interval Confidence.

**Table S3.** Differences in the Hurley system and HS-PGA grades in the adherence to the MD and Ox-LDL in HS patients.

| Adherence to the MD | Grade 1  n=14 | | Grade 2/Grade 3  n=27 | |  |  |
| --- | --- | --- | --- | --- | --- | --- |
|  | **n** | **%** | **n** | **%** | **χ^2^** | *p*-value |
| Low adherence to the MD | 4 | 28.6 | 12 | 44.4 | 3.81 | 0.050 |
| Average adherence to the MD | 3 | 21.4 | 13 | 48.1 | 6.29 | 0.012 |
| High adherence to the MD | 7 | 50.0 | 2 | 7.4 | 1.99 | 0.157 |
|  | **Mean±SD** | | **Mean±SD** | | *p*-value | |
| PREDIMED score | 8.79 ± 4.17 | | 5.93 ± 2.72 | | 0.012 | |
| Ox-LDL | 396.71 ± 213.99 | | 531.00 ± 188.68 | | 0.046 | |

The HS patients with Grade 1 in the Hurley system and HS-PGA had a high adherence to the MD. Skewed variables were back-transformed for presentation in table. Results are expressed as mean ± standard deviation or number/percentage. The chi^2^ (χ^2^) test was used to test the significance of differences between the two groups. Differences between groups were analyzed by unpaired Student’s *t* test. A *p* value in bold type denotes a significant difference (*p <0.05*).

**Abbreviations:** HS, Hidradenitis suppurativa*;* HS-PGA, Hidradenitis suppurativa-Physician’s Global Assessment; MD, Mediterrabnean Diet; ox-LDL, Oxidized low-density Lipoprotein; PREDIMED, PREvención con DIetaMEDiterránea*.*

**Table S4.** Differences of total energy and daily macronutrient/micronutrient intake of HS patients, divided according to the Hurley system and HS-PGA grade.

| Daily intake | Grade 1  n=14 | Grade 2/Grade 3  n=27 | *p* value |
| --- | --- | --- | --- |
| Total energy (kcal) | 2189.43 ± 288.66 | 2329.22 ± 251.81 | 0.117 |
| Protein (gr of total kcal) | 97.19 ± 20.63 | 101.77 ± 16.06 | 0.438 |
| Carbohydrate (gr of total kcal) | 293.76 ± 39.37 | 318.74 ± 37.38 | 0.050 |
| *Complex (gr of total kcal)* | 197.90 ± 26.71 | 214.04 ± 27.99 | 0.082 |
| *Simple (gr of total kcal)* | 98.89 ± 14.84 | 104.70 ± 11.99 | 0.046 |
| Fat (gr of total kcal) | 69.51 ± 7.84 | 71.91 ± 9.44 | 0.394 |
| *SFA (gr of total kcal)* | 24.57 ± 3.10 | 24.81 ± 3.25 | 0.823 |
| *MUFA (gr of total kcal)* | 31.43 ± 3.89 | 32.68 ± 5.14 | 0.392 |
| *PUFA (gr of total kcal)* | 13.51 ± 2.41 | 14.42 ± 2.64 | 0.275 |
| n-6 PUFA (gr/day) | 4.81 ± 2.04 | 6.32 ± 2.92 | 0.062 |
| n-3 PUFA (gr/day) | 8.35 ± 1.66 | 8.28 ± 1.73 | 0.910 |

The HS patients with Grade 1 in the Hurley system and HS-PGA showed the lowest consumption of total and simple carbohydrate compared to grade 2/3. Skewed variables were back-transformed for presentation in the table. Results are expressed as mean ± standard deviation or number/percentage. Differences between groups were analyzed by unpaired Student’s *t* test. A *p* value in bold type denotes a significant difference (*p <0.05*).

**Abbreviations:** HS, Hidradenitis suppurativa*;* HS-PGA, Hidradenitis suppurativa-Physician’s Global Assessment; *SFA*, Saturated Fatty Acids; *MUFA*, MonoUnsaturated Fatty Acids; *PUFA*, PolyUnsaturated Fatty Acids.

**Table S5.** Bivariate proportional odds ratio model to assess the association between total energy and daily macronutrient/micronutrient intake of HS patients, divided according to the Hurley system and HS-PGA grade.

| Parameters | Grade 1  n=14 | | | | Grade 2/Grade 3  n=27 | | | |
| --- | --- | --- | --- | --- | --- | --- | --- | --- |
|  | **OR** | ***p***  **value** | **95%**  **IC** | **R^2^** | **OR** | ***p***  **value** | **95% IC** | **R^2^** |
| Total energy (kcal) | 0.998 | 0.123 | 0.995 – 1.001 | 0.063 | 1.002 | 0.123 | 0.999 – 1.005 | 0.063 |
| Protein (gr of total kcal) | 0.985 | 0.428 | 0.948 – 1.023 | 0.016 | 1.016 | 0.428 | 0.978 – 1.055 | 0.016 |
| Carbohydrate (gr of total kcal) | 0.981 | 0.062 | 0.962 – 1.001 | 0.095 | 1.019 | 0.062 | 0.999 – 1.040 | 0.095 |
| *Complex (gr of total kcal)* | 0.977 | 0.092 | 0.950 – 1.004 | 0.078 | 1.024 | 0.092 | 0.996 – 1.053 | 0.078 |
| *Simple (gr of total kcal)* | 0.946 | 0.054 | 0.895 – 1.001 | 0.099 | 1.057 | 0.054 | 0.999 – 1.117 | 0.099 |
| Fat (gr of total kcal) | 0.968 | 0.412 | 0.896 – 1.046 | 0.017 | 1.033 | 0.412 | 0.956 – 1.116 | 0.017 |
| *SFA (gr of total kcal)* | 0.976 | 0.820 | 0.793 – 1.202 | 0.001 | 1.024 | 0.820 | 0.832 – 1.261 | 0.001 |
| *MUFA (gr of total kcal)* | 0.941 | 0.424 | 0.812 – 1.092 | 0.016 | 1.062 | 0.424 | 0.916 – 1.232 | 0.016 |
| *PUFA (gr of total kcal)* | 0.860 | 0.282 | 0.653 – 1.132 | 0.030 | 1.163 | 0.282 | 0.883 – 1.532 | 0.030 |
| n-6 PUFA (gr/day) | 0.797 | 0.098 | 0.609 – 1.043 | 0.072 | 1.255 | 0.098 | 0.959 – 1.642 | 0.072 |
| n-3 PUFA (gr/day) | 1.023 | 0.908 | 0.694 – 1.507 | 0.000 | 0.978 | 0.908 | 0.664 – 1.440 | 0.000 |

There are no differences in total energy and daily macronutrient/micronutrient intake of HS patients, in grade 1 *vs* grade 2 according to the Hurley system and HS-PGA grade. A *p* value in bold type denotes a significant difference (*p <0.05*).

**Abbreviations:** HS, Hidradenitis suppurativa*;* HS-PGA, Hidradenitis suppurativa-Physician’s Global Assessment; *SFA*, Saturated Fatty Acids; *MUFA*, MonoUnsaturated Fatty Acids; *PUFA*, PolyUnsaturated Fatty Acids.
